# HLA-C*04:01 Affects HLA Class I Heterozygosity and Predicted Affinity to SARS-CoV-2 Peptides, and in Combination With Age and Sex of Armenian Patients Contributes to COVID-19 Severity

**DOI:** 10.3389/fimmu.2022.769900

**Published:** 2022-02-03

**Authors:** Anahit Hovhannisyan, Vergine Madelian, Sevak Avagyan, Mihran Nazaretyan, Armine Hyussyan, Alina Sirunyan, Rubina Arakelyan, Zorayr Manukyan, Levon Yepiskoposyan, Karine R. Mayilyan, Frieda Jordan

**Affiliations:** ^1^ Institute of Molecular Biology, National Academy of Sciences, Yerevan, Armenia; ^2^ Russian-Armenian University, Yerevan, Armenia; ^3^ Armenian Bone Marrow Donor Registry Charitable Trust, Yerevan, Armenia; ^4^ ClinSoft Armenia, Yerevan, Armenia; ^5^ ClinStat Group, Lexington, MA, United States

**Keywords:** COVID-19 severity, HLA classical genes, Armenian population, HLA-I heterozygosity, affinity to SARS-CoV-2, HLA-C*04:01, HLA-B*51:01, severity risk modelling

## Abstract

The novel SARS-CoV-2 coronavirus infection has become a global health concern, causing the COVID-19 pandemic. The disease symptoms and outcomes depend on the host immunity, in which the human leukocyte antigen (HLA) molecules play a distinct role. The HLA alleles have an inter-population variability, and understanding their link to the COVID-19 in an ethnically distinct population may contribute to personalized medicine. The present study aimed at detecting associations between common HLA alleles and COVID-19 susceptibility and severity in Armenians. In 299 COVID-19 patients (75 asymptomatic, 102 mild/moderate, 122 severe), the association between disease severity and classic HLA-I and II loci was examined. We found that the advanced age, male sex of patients, and sex and age interaction significantly contributed to the severity of the disease. We observed that an age-dependent effect of HLA-B*51:01 carriage [odds ratio (OR)=0.48 (0.28-0.80), P_bonf_ <0.036] is protective against severe COVID-19. Contrary, the HLA-C*04:01 allele, in a dose-dependent manner, was associated with a significant increase in the disease severity [OR (95% CI) =1.73 (1.20-2.49), P_bonf_ <0.021] and an advancing age (P<0.013). The link between HLA-C*04:01 and age was secondary to a stronger association between HLA-C*04:01 and disease severity. However, HLA-C*04:01 exerted a sex-dependent differential distribution between clinical subgroups [females: P<0.0012; males: P=0.48]. The comparison of HLA-C*04:01 frequency between subgroups and 2,781 Armenian controls revealed a significant incidence of HLA-C*04:01 deficiency in asymptomatic COVID-19. HLA-C*04:01 homozygous genotype in patients blueprinted a decrease in heterozygosity of HLA-B and HLA class-I loci. In HLA-C*04:01 carriers, these changes translated to the SARS-CoV-2 peptide presentation predicted inefficacy by HLA-C and HLA class-I molecules, simultaneously enhancing the appropriate HLA-B potency. In patients with clinical manifestation, due to the high prevalence of HLA-C*04:01, these effects provided a decrease of the HLA class-I heterozygosity and an ability to recognize SARS-CoV-2 peptides. Based on our observations, we developed a prediction model involving demographic variables and HLA-C*04:01 allele for the identification of potential cases with the risk of hospitalization (the area under the curve (AUC) = 86.2%) or severe COVID-19 (AUC =71%).

## Introduction

The novel coronavirus disease COVID-19, caused by severe acute respiratory syndrome coronavirus 2 (SARS-CoV-2), has emerged at unprecedented scale and ferocity, putting enormous pressure on health systems globally. As of 31 August 2021, according to the World Health Organization, the cumulative number of coronavirus-infected patients identified is 216,867,420 (1). In terms of the clinical course of the disease, its spectrum can range from asymptomatic to severe manifestation, with acute respiratory failure leading to death in some cases (2, 3). The early reports on the COVID-19 clinical characteristics indicate that up to 20% of patients required admission to critical care, such as intensive care unit (ICU), and prolonged support by mechanical ventilation in case of respiratory failure. In-hospital mortality rate was as high as 28% (3, 4). Preceding health conditions, age and sex of individuals, environmental and socioeconomic factors are known to affect both the susceptibility and the severity of COVID-19 (5, 6). Meanwhile, knowledge on the influence of the host genetic profile on the susceptibility and outcome of the disease is being continuously generated.

HLA molecules play a pivotal role in the development and regulation of immune response and are shown to be associated with the predisposition to multiple viral diseases, such as HIV (7, 8), HBV, HCV (9), CMV (10), and SARS-CoV-1 (11–14). Several studies have reported a link between HLA alleles and COVID-19 infection in different populations (15–20), some of which also indicated the association between an HLA-C allele and the severe clinical course of the disease (17, 19, 21, 22). Nevertheless, our understanding of the HLA genetic contribution to the COVID-19 clinical manifestation and severity remains insufficient and requires further exploration. Furthermore, because of HLA genetic diversity across human populations, the association between the disease severity and specific HLA alleles may vary from one ethnic/ancestral group to another. Therefore, HLA landscape of each population should be also considered in COVID-19 association studies (23).

Here, we report the results of the HLA classic loci association analyses with severity of COVID-19 infection in the Armenians, a population with its unique HLA variations developed over long-term genetic isolation (24). Our findings implicate the HLA-C*04:01 allele in the severe course of the disease in a dose-dependent manner, and the HLA-B*51:01 carriage in the protection against the disease but with a lesser extent.

## Materials and Methods

### Subjects

The study included 299 SARS-CoV-2 infected patients. During April-May 2020, blood and nasopharyngeal swab samples from patients were collected in six hospitals of the Health Ministry of the Republic of Armenia: National Center for Infection Diseases, Surb Grigor Lusavorich, Surb Astvatsamayr and Artashat Medical Centres, Maralik and Akhuryan Mother and Child Health Centers. The diagnosis was performed on nasopharyngeal specimens by detection of SARS-CoV-2 RNA, using the following kits: SARS-CoV-2/SARS-CoV Multiplex REAL-TIME PCR Detection Kit (LLC “NPF DNA-Technology”, Cat. No. R3-P436-23/9EU), LiliF™ COVID-19 Real-time RT-PCR Kit (iNtRON Biotechnology, Cat. No. IPH21505.50) and Real-time-fluorescent-RT-PCR (BGI, Cat. No. HW5105). Patients were classified into three groups according to their disease symptoms. The severe disease group (n=122) comprised patients with severe respiratory problems, who required critical care at ICU and the use of oxygen supplementation and/or mechanical ventilation. In the second group, namely mild (in figures) symptomatic group (n=102), were included patients hospitalized during the same period with various mild and moderate COVID-19 symptoms, such as fever, loss of taste, body aches, fatigue, etc., but not requiring ICU admission. The asymptomatic group (n=75) consisted of hospital staff at the COVID-19 treating centers who tested positive for the virus but had no symptoms.

To compare the allelic frequency profiles of patient groups with those specific to the general population, we used HLA data of 2,781 Armenian individuals who were registered donors at the Armenian Bone Marrow Donor Registry (ABMDR) before the pandemic. This group was used as a control cohort for age-genotype and allele frequency analyses. Informed consent for genetic studies was obtained from all subjects. The study protocol was approved by the Ethics Committee of Yerevan State Medical University after Mkhitar Heratsi (Meeting of 04.06.2020, Protocol N 7-3/20), and carried out following the Declaration of Helsinki.

### HLA Sequencing

Blood samples (3 ml, EDTA) were sent out to HistoGenetics LLC laboratory (Ossining, NY) for HLA sequencing of HLA-A, HLA-B, HLA-C, HLA-DRB1, HLA-DQA1, HLA-DQB1, HLA-DPA1, and HLA-DPB1 loci (25). Next-generation sequencing was performed using Illumina MiSeq and PacBio platforms.

### The SARS-CoV-2 Peptides Binding Affinity by HLA Class I Molecules

A number of SARS-CoV-2 peptides binding ability for each HLA-A, HLA-B, HLA-C, and total HLA class I molecules was calculated from the data provided by Nguyen et al. (26). Based on the availability of HLA alleles represented in the Armenian population and, particularly, in this dataset, we were able to predict the number of SARS-CoV-2 peptides binding to the HLA-A for 281, HLA-A for 276, and HLA-C and HLA class I molecules for 234 and 203 patients, respectively.

### Statistics

A Hardy-Weinberg equilibrium (HWE) of HLA genotypes was calculated with PyPop (Python for Population genomics) v.0.7.0 software (27). The HLA allele frequency data for world populations were obtained from the Allele Frequency Net Database (28).

Statistical analyses were performed using SPSS program v. 17.0 and STATISTICA v.6.1 StatSoft Inc. (Tulsa, USA). For general statistics, Fisher’s exact, χ2 or logistic regression tests were used for comparison of frequencies. Kolmogorov-Smirnov test with Lilliefors correction was applied for inspection of normality of the continuous data distribution. Spearman’s rank or Pearson’s r tests were used for correlation analyses based on the data distribution. Two-tailed Student’s t and Mann-Whitney tests were used for comparison of two groups, while ANOVA and Kruskal–Wallis tests were used for multiple groups based on the distribution of a continuous variable. Linear regression analysis was applied for continuous variable(s) to test the difference between several groups, and/or for conditional analysis of covariates. In a linear regression of the HLA class I affinity to the SARS-CoV-2 peptides, four outliers were omitted, as they had a value more than 1.5 times the interquartile range from either 25th percentile or 75th percentile of the group.

To test the association of age and sex with the COVID-19 severity characteristics as well as with the HLA allele genotypes, unconditional linear and ordinal logistic regression analyses were performed, respectively. For association analyses of each common HLA allele (freq > 0.05%) with disease severity, unconditional ordinal logistic regressions were applied in additive mode:


$$ln(OR)∼\beta_{HLA}*dose+\theta_{asymptomatic}+\theta_{mild}$$

where *dose* is the number of the HLA allele of interest in the genotype of the given HLA locus (or the number of diverse HLA class I alleles in all three loci, when testing the effect of HLA class I heterozygosity). Thus, allelic-doses 0, 1, and 2 correspond to genotype with homozygote absence, heterozygote and homozygote presence of a given HLA allele (i.e., HLA genotypes: 0/0, 0/1, and 1/1, respectively), while for HLA class I heterozygosity number of alleles ranged between 3 (homozygote for all three HLA class I loci) and 6 (heterozygote for HLA-A, -B and –C loci). P values of nominally significant associations were adjusted for multiple comparisons using Bonferroni correction (P_bonf_). Since age and sex, as well as sex and age interaction were significantly different between subgroups of cases, subsequent conditional ordinal logistic regression analyses were performed with the following model:


$$\begin{array}{l} ln(OR)∼\beta_{age}*age+\beta_{sex}*sex\\ +\beta_{interaction}*(age*sex)+\beta_{HLA}*dose\\ +\beta_{interaction}*(dose*sex)+\theta_{asymptomatic}+\theta_{mild} \end{array}$$

where *OR* is calculated from the probability of having given or much severe course of disease, *dose* is the number of the HLA allele of interest in the genotype of the given HLA locus, *θasymptomatic* is β of the first order intercept and *θmild* is β of the second order intercept. The models fit were assessed by Akaike`s information Criterion (AIC) (29) and Bayesian information Criterion (BIC). In case of HLA-C*04:01 these variables were 556.9 and 582.4, respectively, while for HLA-B*51:01 carriage – 561.0 and 586.9, respectively. For the contextualization of potential genetic mechanisms of HLA allele association with the COVID-19 severity, the allelic frequencies in asymptomatic, mild/moderate, and severe patient groups were compared with those of the controls from the general population applying the χ2 test.

HLA haplotype prediction and their association with the disease manifestation characteristics were performed using Bringing ImmunoGenomics Data Analysis Workflow Gaps (BIGDAWG) R package (30).

We developed a risk prediction model for detecting severe COVID-19 cases admitted to ICU, as well as for the identification of hospitalized vs. non-hospitalized COVID-19 cases. For this, we merged a risk prediction models of unconditional ordinal logistic regressions of severity characteristics against demographic and genetic predictors. The final association model was the following:


$$\begin{array}{l} ln(OR)∼\beta_{male\;vs.\;female}*sex+\beta_{age(male)}*age\\ +\beta_{age(female)}*age+\beta_{HLA}*dose \end{array}$$

where *OR* is calculated from the probability of having given or much severe COVID-19 using the following coefficients: β_male vs. female_ =2.300; β_age(male)_ = 0.049 per year; β_age(female)_ = 0.091 per year; β_HLA_=0.545 per copy of HLA-C*04:01 allele in genotype.

The receiver operating characteristics (ROC) curve was plotted using SPSS program v.17.0. The area under the curve (AUC) was used to evaluate the ability of the model to discriminate between severe and non-severe cases as well as between asymptomatic and cases with clinical symptoms of COVID-19 disease.

## Results

### Demographic Characteristics of Patients

The demographic characteristics of the controls and the patients from each group are summarized in **Table 1**. The huge disparity across cohorts in demographics was based on the diverse aim in sample collection (i.e. relatively young controls were registered donors at the Armenian Bone Marrow Donor Registry), the hidden course of the disease in asymptomatic cases (all asymptomatic cases in the study were hospital workers of non-retiremental age with the majority of female staff members, who routinely underwent SARS-CoV-2 RNA test) and the well-documented age association of COVID-19 severity. Particularly, in unconditional ordinal logistic regression analyses, the severity score of COVID-19 infection was found to be significantly correlated with the advanced age (P<9.5E-18; see **Figure S1A**) but not to the sex of the patients (P>0.87). Despite the latter non-significant association, sex of individuals showed disparity across clinical subgroups (χ2 = 8.5; P<0.014) (**Figure S1B**). Apparently, there was a significant age and sex interaction as well. Particularly, when age and sex interaction term was added to the model, all the three variables, i.e., age (P<1E-20), sex, and age and sex interaction were significantly associated with severity score, which unveiled ~10-fold higher relative OR for males versus females: [OR ± 95% confidence interval (CI) = 9.98 (1.66-59.97), P<0.012] (**Table S1**). OR of age effect per year in females versus males was equal to 1.042 (95% CI: 1.011-1.075, P<0.0075).

**Table 1:** Demographic characteristics of patient subgroups and controls.

	Control(n = 2,781)	Asymptomatic(n = 75)	Mild/Moderate(n = 101)	Severe(n = 122)	P-value
**Age, M ± SD**	33.17 ± 9.67	40.73 ± 14.56	60.32 ± 13.85	63.57 ± 12.11	0.0001
**Sex, male, n (%)**	1529 (55)	27 (36)	57 (56.4)	49 (40)	0.87

### Associations of HLA Alleles With Susceptibility and Severity of COVID-19

In the total group of patients, out of the eight investigated loci, only the HLA-A genotype was subtly departed from the HWE (P<0.048). Using data of 299 patients and 2,781 population control subjects, we first checked for the association of HLA common alleles with susceptibility to COVID-19, and did not find any significant association (**Table S2**).

Three HLA alleles, HLA-C*04:01 (P<0.0035), B*35:01 (P<0.047), and HLA-B*51:01 (P<0.0075) were nominally associated with the disease severity in an unconditional additive mode (**Table 2**). After applying Bonferroni’s correction, only the association of the HLA-C*04:01 allele remained significant (P_bonf_<0.021), while HLA-B*51:01 showed a trend for an association. None of the two and three allelic common haplotypes showed a significant association with the disease severity characteristics (data not shown), indicating an independent nature of HLA-C*04:01 and HLA-B*51:01 associations.

**Table 2:** Unconditional ordinal logistic regressions for the association of the genotypes of each common allele (freq > 0.05%) of the HLA class I and class II loci with the disease severity characteristics in the additive model of each common allele.

Loci	Alleles	Frequency	OR(±95% CI)	P-value	P_bonf_
A	02:01	0.16	1.12 (0.74 - 1.70)	0.60	NS
A	24:02	0.14	1.18 (0.77 - 1.82)	0.45	NS
A	01:01	0.10	1.12 (0.69 - 1.81)	0.64	NS
A	03:01	0.09	0.64 (0.39 - 1.06)	0.085	NS
A	11:01	0.07	0.71 (0.38 - 1.31)	0.27	NS
A	03:02	0.07	1,23 (0.68 - 2.23)	0.49	NS
A	26:01	0.05	1.34 (0.70 - 2.56)	0.39	NS
A	binned	0.32	0.98 (0.72 - 1.33)	0.88	NS
*B*	*51:01*	*0.11*	*0.51 (0.32 - 0.84)*	*0.0075*	*0.06*
*B*	*35:01*	*0.08*	*1.82 (1.01 - 3.28)*	*0.047*	*NS*
B	44:02	0.07	1.20 (0.69 - 2.13)	0.51	NS
B	18:01	0.07	0.98 (0.53 - 1.81)	0.94	NS
B	49:01	0.06	1.30 (0,71 - 2.39)	0.39	NS
B	38:01	0.05	1.20 (0,60 - 2.41)	0.60	NS
B	50:01	0.05	0.98 (0.50 - 1.90)	0.95	NS
B	binned	0.51	0.95 (0.70 - 1.29)	0.74	NS
**C**	**04:01**	**0.20**	**1.72 (1.20 - 2.49)**	**0.0035**	**0.025**
C	07:01	0.12	1.23 (0.79 - 1.91)	0.36	NS
C	12:03	0.11	0,91 (0,57 - 1.45)	0.68	NS
C	06:02	0.11	0.97 (0.60 - 1.56)	0.90	NS
C	07:02	0.07	1.10 (0.62 - 1.97)	0.74	NS
C	16:04	0.05	1 (0.52 - 1.89)	0.99	NS
**C**	**binned**	**0.35**	**0.63 (0.47 - 0.86)**	**0.0032**	**0.022**
DRB1	11:04	0.16	1,25 (0.82 - 1.91)	0.29	NS
DRB1	11:01	0.09	0.88 (0.53 - 1.46)	0.61	NS
DRB1	07:01	0.08	1.37 (0.78 - 2.39)	0.27	NS
DRB1	03:01	0.07	1.61 (0.89 - 2.89)	0.11	NS
DRB1	04:04	0.06	1.30 (0.66 - 2.58)	0.45	NS
DRB1	04:02	0.05	0.69 (0.37 - 1.29)	0.25	NS
DRB1	04:03	0.05	0.76 (0.41 - 1.39)	0.37	NS
DRB1	15:01	0.05	1.10 (0.60 - 1.99)	0.76	NS
DRB1	binned	0.38	0.80 (0.59 - 1.09)	0.15	NS
DQA1	05:05	0.30	1.01 (0.73 - 1.41)	0.93	NS
DQA1	03:01	0.16	0.78 (0.53 - 1.15)	0.21	NS
DQA1	01:02	0.10	1.33 (0.83 - 2.14)	0.24	NS
DQA1	02:01	0.08	1.37 (0.78 - 2.37)	0.27	NS
DQA1	01:01	0.08	1.02 (0.57 - 1.82)	0.95	NS
DQA1	05:01	0.07	1.61 (0.89 - 2.89)	0.11	NS
DQA1	01:04	0.07	0.56 (0.31 - 1.03)	0.06	NS
DQA1	03:03	0.06	0.78 (0.42 - 1.43)	0.42	NS
DQA1	01:03	0.05	1.29 (0.65 - 2.56)	0.47	NS
DQA1	binned	0.03	1.40 (0.97 - 2.02)	0.076	NS
DQB1	03:01	0.32	0.94 (0.68 - 1.29)	0.70	NS
DQB1	03:02	0.16	0.77 (0,52 - 1.14)	0.19	NS
DQB1	05:01	0.08	0.88 (0.50 - 1.53)	0.65	NS
DQB1	02:01	0.07	1.61 (0.89 - 2.89)	0.11	NS
DQB1	02:02	0.07	1.21 (0.65 - 2.24)	0.56	NS
DQB1	05:03	0.07	0.56 (0.31 - 1.03)	0.061	NS
DQA1	binned	0.22	0.68 (0.29 - 1.59)	0.37	NS
DPA1	01:03	0.79	0.92 (0.64 - 1.32)	0.65	NS
DPA1	02:01	0.14	1.35 (0.89 - 2.04)	0.16	NS
DPA1	binned	0.07	1.27 (0.91 - 1.75)	0.16	NS
DPB1	04:01	0.40	0.82 (0.61 - 1.10)	0.18	NS
DPB1	04:02	0.17	0.91 (0.61 - 1.34)	0.62	NS
DPB1	02:01	0.15	1.17 (0.77 - 1.77)	0.46	NS
DPB1	binned	0.27	0.63 (0.33 - 1.19)	0.16	NS

The HLA alleles highlighted in bold and in italic fonts correspond to the association with COVID-19 severity at P <0.05 and 0.05< P<0.1 levels, respectively. NS denotes a non-significant association after Bonferroni correction for multiple testing.

**Figure 1:**
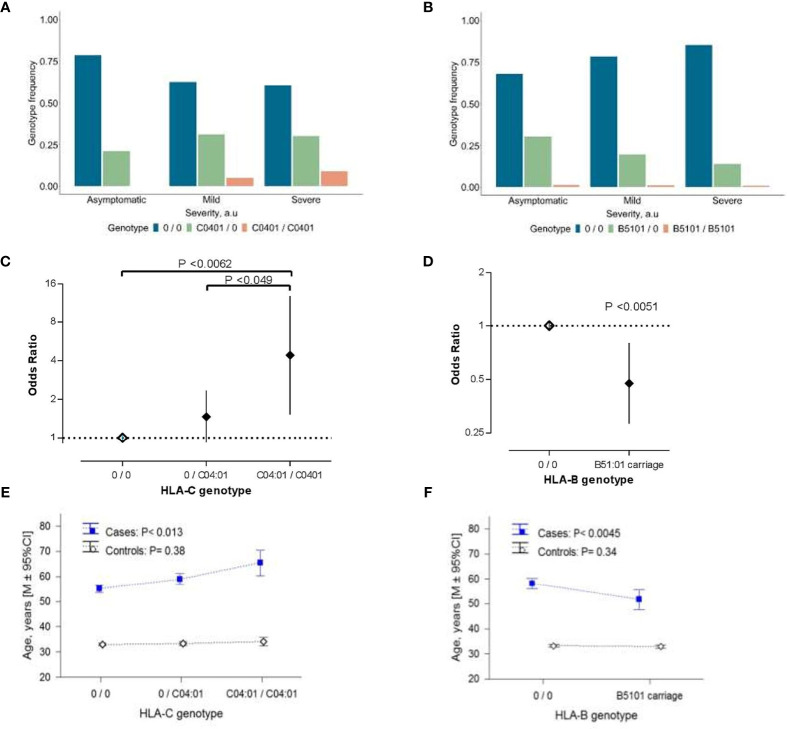
The association of HLA class I alleles with COVID-19 severity. **(A, B)** Allelic distribution of HLA-C*04:01 **(A)** and HLA-B*51:01 **(B)** in COVID-19 patient subgroups with asymptomatic (n=75), mild/moderate (n=102), severe (n=122) manifestation of the disease. **(C, D)** COVID-19 disease severity risk associated odds ratio (OR) of HLA-C*04:01 **(C)** and HLA-B*51:01 **(D)** variations in the ordinal logistic regression analyses. Error bars represent 95% confidence intervals (95% CI) of OR. Data of HLA-C genotypes are the result of the ordinal logistic regression analyses using codominant model for visualization purposes. **(E, F)** Age distribution in the COVID-19 patients and controls from the general population associated with the allelic-load of HLA-C*04:01 **(E)** and HLA-B*51:01 carriage **(F)**. In **(E, F)** P-values were derived from ANOVA tests to analyze differential genotype effect separately in cases and controls. In **(C–F)** “0” denotes any other allele different from the allele of interest. In **(E, F)** control groups refers to 2,781 individuals who were registered donors at the Armenian Bone Marrow Donor Registry (ABMDR) before the pandemic.

Patients homozygous for the HLA-C*04:01 allele were identified in the mild/moderate and severe subgroups (**Figure 1A**), which resulted in a dose-dependent increase of the risk for severe clinical manifestation (**Figure 1C**). In contrast, only one person from each subgroup was homozygote for HLA-B*51:01 (**Figure 1B**), causing an inflation of statistical error. Therefore, we applied a dominant model for reinspection of this association. Our results indicated that the presence of HLA-B*51:01 allele conferred protection against the severe clinical manifestation of COVID-19 infection (P<0.0051; **Figure 1D**), which survived the correction for multiple testing (P_bonf_ <0.036).

HLA-C*04:01 exerted a sex-dependent differential distribution between subgroups of COVID-19 cases. Particularly, in females but not in males [HLA-C*04:01 per copy: OR (95% CI) = 1.21(0.72-2.02), P =0.48], carrying this allele, appeared to predispose to a relatively severe outcome in dose-dependent manner [HLA-C*04:01 per copy in females: OR (95% CI) = 2.15 (1.44-4.39), P = 0.0012] (**Figure S2**). Besides, we observed an association of the allelic-load of HLA-C*04:01 and the presence of HLA-B*51:01 with the age of patients (P<0.013 and P<0.0045, respectively; **Figures 1E, F**). Particularly, the relationship between the age and HLA-C*04:01 mirrored the association between advancing age and the severity score of COVID-19 (**Table 1**), while that of HLA-B*51:01 was in the opposite direction. We additionally performed the conditional analyses controlling for the age and sex of the patients. Unlike HLA-B*51:01 (P>0.15), the relationship between HLA-C*04:01 and the disease severity did not meaningfully change, reaching the significance threshold of suggestive association (P<0.063). Notably, mirroring a sex-related differential distribution between clinical subgroups, the HLA-C*04:01 dose-dependent association remained significant in females (P<0.014) but not in male patients (P=0.96; see **Table S3**). We found that the association of HLA-C*04:01 genotype with the age in the COVID-19 cases was secondary to the association between the genotype and the COVID-19 severity characteristics (HLA-C*04:01 association with age conditioned for the disease severity, P=0.13). Moreover, a similar analysis in the control cohort with 2,781 individuals from the general population showed no correlation between allelic-load of HLA-C*04:01 or HLA*B51:01 and age (**Figures 1E, F**). Thus, HLA-C*04:01 association with advancing age was specific to the patients and rather evolved from the allele precipitation in subgroups of mild/moderate and severe COVID-19 cases with a relatively higher average age (**Table 1** and **Figure S1A**).

To elucidate the association mechanism of the HLA-C and COVID-19 severity, we compared the frequency distribution of the HLA-C*04:01 allele in the three clinical subgroups with a representative cohort of the Armenian population. In comparison with controls, a non-significant difference in HLA-C*04:01 allele frequency was observed for the COVID-19 patients with mild/moderate (P=0.93) and severe (P=0.15) symptoms composing the group of hospitalized inpatients (**Figure S3A**). In contrast, asymptomatic patients with positive SARS-CoV-2 had significantly lower HLA-C*04:01 frequency (P<0.012). Besides, we found that the frequency of HLA-B*51:01 in discrete subsets of patients was not significantly different from that of the general population (0.063 ≤ P < 0.73; **Figure S3B**). Thus, our results are consistent across allelic and genotype analyses and indicate that the lack of HLA-C*04:01 is protective against a manifestation of the disease symptoms in Armenian patients rather than its load confers a severe course of the disease.

### Testing HLA Class I-Related Theories on COVID-19 Severity

**Figure 2:**
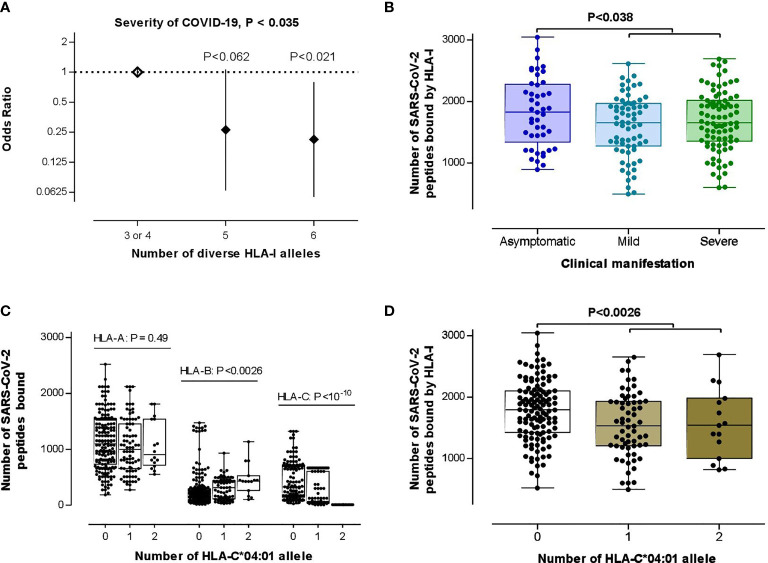
Testing the theories on the association between HLA class I loci and COVID-19 severity. **(A)** HLA class I heterozygosity was a predictor of the disease severity. Error bars represent 95% confidence intervals (95% CI) of OR. **(B)** Differential HLA class I affinity to SARS-CoV-2 peptides was observed in asymptomatic patients (n=45) compared with mild/moderate (n=67) to severe (n=87) cases (P-value was driven by two-tailed t-test) for whom affinity data from the recent *in silico* study were available (26). **(C)** Effect of HLA-C*04:01 allelic load on HLA-A (n=281), HLA-B (n=276), HLA-C (n=234) binding capacity to SARS-CoV-2 peptides (P-value was driven by Kruskal–Wallis test). **(D)** HLA-C*04:01 allele carriage was associated with an attenuated average HLA class I binding affinity to SARS-CoV-2 peptides (P-value was driven by a two-tailed t-test of the data from 78 carriers vs 121 non-carriers). In **(B–D)**, box-plots indicate median and interquartile ranges of data distribution without four outliers.

Searching for the biological consequences of detected HLA-C*04:01 association with clinical manifestation, we tested a possible contribution of this allele to the HLA class I heterozygosity and number of SARS-CoV-2 peptides presenting ability, which reportedly are protective against progression of COVID-19 (31). For this goal, we first tested if those HLA class I variables significantly correlate with COVID-19 severity characteristics in this data set. Even though there was no statistical differences for separate HLA-A, HLA-B and HLA-C loci between subgroups (P=0.25, P=0.48, P=0.23, respectively, for HLA-A, -B, and -C), we observed a significant association between low HLA class I heterozygosity and the disease severity [unconditional likelihood ratio test global P< 0.035; **Figure 2A**], which overcame the covarying effect of age and sex [global P<0.037, in comparison with heterozygote genotype for 0 or 1 HLA class I locus, heterozygotes for two HLA class I loci: OR (95% CI) = 0.23 (0.05-1.025), P=0.054; heterozygotes for all three HLA class I loci: OR (95% CI) = 0.19 (0.046-0.77), P = 0.020]. While testing the hypothesis on the association between COVID-19 severity and predicted low number of HLA-recognizable SARS-CoV-2 peptides (26, 31), HLA-A, HLA-B and HLA-C loci showed no difference between subgroups (Mann-Whitney test P>0.24; see **Figures S4A–C**). Thus, we could not confirm the findings of Iturrieta-Zuazo et al. (31) on separate HLA class I molecules. As to the general HLA class I affinity, we found a better theoretical capacity to bind SARS-CoV-2 peptides in asymptomatic patients compared to mild/moderate and severe cases (**Figure 2B**).

Strikingly, both of those HLA class I variables, i.e., heterozygosity and SARS-CoV-2 peptide presenting ability, showed stronger associations to the HLA-C*04:01 allele than to the clinical manifestation of COVID-19. First, the HLA-C*04:01 homozygosis was simultaneously associated with low HLA class I and HLA-B heterozygosity (P<4.2E-12 and P<0.0039, respectively; **Figure S5**). Second, the allelic-load of HLA-C*04:01 increased and decreased, respectively, the median number of SARS-CoV-2 peptides affinity to HLA-B and HLA-C molecules (**Figure 2C**). Lastly, those counter-effects of HLA-C*04:01-load culminated in a relatively low HLA class I-binding ability in the HLA-C*04:01 carriers (**Figure 2D**).

As in other populations, the LD between HLA-B and HLA-C loci was the highest across HLA classical genes in our sample (R^2 =^ 0.98). To test if any HLA-C allele homozygosity is a sufficient genetic determinant to drive HLA I and HLA-B low heterozygosity observed in severe COVID-19 due to the high LD between HLA-B and HLA-C, we have performed similar analyses with HLA-C*07:01 (next frequent HLA-C allele in our cohort, see **Table 2**). We found that although HLA-C*07:01 homozygosity significantly affects HLA-B heterozygosity (P<0.043; cf. HLA-C*04:01: P<0.0056), but in overall statistics, this HLA-C*07:01 effect is not significant (P=0.13; cf. HLA-C*04:01: P<0.0039, see **Figure S5B**). Besides, due to relatively low frequency than HLA-C*04:01, and non-significant association with COVID-19 (P>0.36), it does not preclude a significant association between a scanty HLA class I heterozygosity and COVID-19 (after controlling for HLA-C*07:01, P<0.035). These comparative data indicate that the association between HLA-C*04:01 and COVID-19 is so substantial that it also drives a decrease in HLA I heterozygosity in severe cases.

In conjunction with the results of an *in silico* analysis that the HLA-C*04:01 allele is one of the least efficient for SARS-CoV-2 peptide presentation (26), our aforementioned findings suggested that the scanty HLA class I heterozygosity and SARS-CoV-2 peptide presenting ability in COVID-19 cases with clinical symptoms may be driven by the stronger association of the HLA-C*04:01 with the disease severity. Therefore, we performed a couple of reciprocal conditional analyses. In an ordinal logistic regression, we found that the HLA-C*04:01 association with COVID-19 severity retained its significance (P<0.033), despite controlling for HLA class I heterozygosity. On the other hand, the significant diversity in the SARS-CoV-2 peptide presenting ability of the HLA class I molecules across severity groups was constrained (P>0.17). Simultaneously, HLA-B (P<0.030), HLA-C (P<3.3E-07) and HLA class I (P<0.0038) binding ability showed a similar pattern of the association to the HLA-C*04:01 allelic–load, as in unconditional analyses. These results implicate HLA-C*04:01 allele in COVID-19 severity with a biological consequence affecting HLA class I qualitative and quantitative properties, and serving as a genetic determinant for the SARS-CoV-2 peptide presentation quantitative trait (QT) beyond its effect on the HLA-C locus.

### Predictors of Hospitalization and ICU Admission of Armenian COVID-19

**Figure 3:**
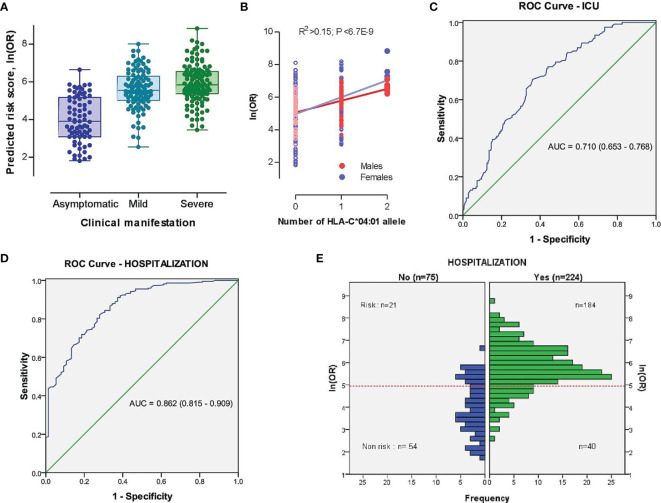
The model characteristics for prediction of the risk scores between severe (ICU-admitted) and non-severe, as well as hospitalized and non-hospitalized COVID-19 cases. **(A)** Distribution of risk scores in different subsets of COVID-19 cases based on the clinical picture. **(B)** Distribution of risk scores in different subsets of COVID-19 cases based on the genomic number of HLA-C*04:01 allele. P-value was driven from Pearson correlation test. **(C, D)** Receiver operating characteristic (ROC) curve involving the contribution of HLA-C*04:01, sex, and sex-dependent age effects for discrimination of severe vs. non-severe **(C)** and hospitalized vs. non-hospitalized **(D)** COVID-19 cases. True positive rate (sensitivity) was plotted against false positive rate (1-specificity). The areas under the curves are presented for each outcome. **(E)** Histogram plot of hospitalized and non-hospitalized cases versus distribution of ln(OR). The dotted red line represents the threshold of the plot obtained from the AUC (Area Under Curve) with optimal sensitivity (0.821) and specificity (0.720). A significant difference was observed between risk and non-risk cases (OR (95% CI) = 11.83 [6.43-21.75], P=6,54E-21).

Based on our findings, we further developed a risk prediction model for detecting severe COVID-19 cases admitted to the ICU, as well as for the identification of hospitalized vs. non-hospitalized COVID-19 cases by merging risk prediction models of unconditional ordinal logistic regressions of severity characteristics against demographic (i.e., age, sex, and sex and age interaction) and HLA-C*04:01 predictors (see *Material and Methods*). The risk scores better separated hospitalized patients with mild to severe symptoms from asymptomatic COVID-19 subjects than severe versus non-severe cases (**Figure 3A**), as the individual age was the dominant variable (**Figure S6**). Nevertheless, the contribution of HLA-C*04:01 allele explained 15.4% of the variation in log-transformed OR score (**Figure 3B**). Thus, the AUC of a risk prediction model for a severe course of the disease that includes HLA-C*04:01, age, sex, and sex and age interaction was 0.710 (95% CI = 0.653-0.768) with a significant difference between severe and non-severe cases (P=6,26E-10; **Figure 3C** and **Figure S7**). The resulting OR for predicting severe cases or cases admitted to ICU was relatively moderate [OR=4.22 (2.57-6.92); sensitivity = 0.705, specificity = 0.638]. The same model, however, was more efficient in indicating hospitalization of cases with AUC equal to 0.862 (95% CI =0.815-0.909), with a sensitivity of 0.821 and specificity of 0.720. The threshold of the plot was calculated from the optimal sensitivity and specificity in the AUC, which revealed a significant difference between hospitalized and non-hospitalized cases (P=6,54E-21; **Figure 3D**). The model was able to discriminate higher OR between hospitalized and non-hospitalized cases [OR (95% CI) = 11.83 (6.43-21.75), **Figure 3E**].

## Discussion

Based on the conjoint approach of HLA classical loci genotype and allelic distribution analyses between Armenian population controls and COVID-19 cases, as well as between patients with different clinical phenotypes, we discovered an age-related protective effect of the HLA-B*51:01 carriage against COVID-19 severity. In contrast, we found that HLA-C*04:01 allelic-load contributes to the risk for the severe COVID-19 independently from age and classical HLA variables. However, the HLA-C*04:01 association with COVID-19 manifestation is rather in concert with female gender and older age. Along with the expected significant decrease in cumulative heterozygosity of HLA class I loci, we report a previously undescribed decrease in heterozygosity of HLA-B locus blueprinted by the HLA-C*04:01 homozygous genotype. These changes in HLA heterogeneity may translate to the SARS-CoV-2 peptide presentation relative inefficacy by HLA-C and HLA class I molecules in HLA-C*04:01 carriers, simultaneously enhancing the appropriate potency of HLA-B. In patients with mild/moderate to severe COVID-19, due to high prevalence of HLA-C*04:01, these effects provide a decrease of the HLA class I loci heterozygosity and a down-modulation of the predicted HLA class I ability to recognize SARS-CoV-2 peptides. Given these findings, we have developed a risk prediction model for the early identification of Armenian COVID-19 cases with a potential to be hospitalized or, subsequently, to be admitted to intensive care units. The genomic number of HLA-C*04:01 allele and demographic variables (i.e. age and sex) compose the model, in which >15% potential share in the detection of cases with adverse clinical phenotypes belongs to HLA-C*04:01.

Our finding on the association between HLA-C*04:01 genomic quantity and COVID-19 severity is consistent with some previous reports of cross-sectional studies, involving Indian (32), German (21) and USA (multiethnic) (21, 22) cohorts. Consistently to our results, Warren and Birol (22) also report that the high prevalence of HLA-C*04:01 allele may predispose to severe COVID-19 in patients with a white ethnic background, female gender and advanced age (≥65 years old). This phenomenon of interaction with sex is not unusual for the HLA and COVID-19 association, as it can appear even on haplotype level as well (33). Nevertheless, most studies land partial or no support to our findings (16, 17, 19, 34, 35). Thus, the first genome-wide association study (GWAS) reports a trend for HLA-C*04:01 association to the disease severity in the Italian population with a marginal significance, and a significantly lower number of HLA class I bound SARS‐CoV‐2 peptides in the most severe group of the Italian and Spanish patients (35). Another study indicates HLA-C*04:01 association with susceptibility but not COVID-19 severity in the Sardinian/Italian population (34). Despite this allelic variability conferring COVID‐19 or its clinical heterogeneity across ethnicities, the inability of the identified alleles to effectively bind the SARS‐CoV‐2 viral peptides and to trigger an adequate immune response among others may be one of the main causative mechanisms for severe disease (20, 26, 31, 32, 36).

HLA class I molecules present antigens to cytotoxic T lymphocytes (CTLs) and natural killer (NK) cells. HLA-C, being the least potent in antigen presentation to CTLs (37), works extremely well in controlling and modulating NK cell immune response (38, 39). The HLA-C mediated protection, harnessing NK cells, is relevant to a number of viral infections (40–42), including SARS‐CoV‐1 infection (43). In spite of genetic association data, a similar role of HLA-C in severe COVID-19 is yet unknown. An impediment for a direct approximation of genetic findings are transcriptional and epigenetic changes seen in severe cases, such as two differentially methylated CpG sites regulating HLA-C locus (44), and HLA-C alternative transcription and RNA splicing events (45). Another obstacle is a high incidence of lymphopenia (46), in particular, the reduced number of NK cells in the peripheral blood of COVID-19 patients, which is related to the severe stage of infection (47–50). Moreover, it is already established that the pathogenesis of severe SARS‐CoV‐2 infection involves a hyper-function of CD4+ and CD8+ T cells and NK cell hyporesponsiveness (49).

Given the key role of HLA-C molecules in antigen presentation to KIR receptors on NK cells, HLA-C*04:01 molecules with (i) a poor affinity to the SARS-CoV-2 peptides (26), (ii) a low cell surface expression ability [due to bearing HLA-C 3UTR’ target site that miR-148a binds (51)], (iii) and differential avidity to its cognate receptors (i.e. high for the inhibiting KIR (KIR2DL1) than the activating KIR [functional KIR2DS4 (52)], may cause NK cell hyporesponsiveness since the early phase of infection (49). As NK cells are at the forefront of antiviral response, particularly at that of the respiratory system (53), NK hyporesponsiveness may delay SARS‐CoV‐2 viral recognition and, subsequently, generate an inadequate immune response, supporting the virus propagation and lung damage, such as seen in severe pneumonia with a genetically predicted HLA-C low expression (54). A series of genetic studies with a Sardinian/Italian sample potentially support this hypothesis, by reporting a significantly high prevalence of both, HLA-C*04:01 allele and NK-cell inhibitor KIR2DL1 receptor, in COVID-19 cases (34, 55).

As the avidity of KIR2DS4 receptor is limited to several HLA class I alleles (52), the other mechanism may involve HLA-C*04:01 antigen presentation to this receptor. At the late phase of the COVID-19 progression, in the frame of cytokine storm and lymphopenia (46, 56), the lung residing infected monocytes might recruit a new portion of NK cells from blood (48). The specific induction of HLA-C expression on differentiated macrophages in the lung of COVID-19 HLA-C*04:01 carriers (57) may trigger CTL and NK cells activation *via* KIR2DS4 (52), and result in those cells exhaustion. Of note, the two parts of our hypothesis are not mutually exclusive and rather discriminate differential spatial and temporal responses of the HLA-C restricted CTL and NK cells. Remarkably, considerable literature is supportive of the latter interpretation, as several reports from different populations with high prevalence of the functional KIR2DS4*001 gene (58, 59) delineate a risk association of HLA-A*11:01, HLA-C*05:01, HLA-C*04:01, and HLA-C*16:01 to the severe or fatal COVID-19 (16, 17, 19, 21, 22, 34, 60–64), and all of those alleles are recognized by the functional KIR2DS4. From the other side of the same coin, HLA-B enhanced affinity to the SARS-CoV-2 viral peptides, coinciding with the carriage of HLA-C*04:01, may over-activate CD8+ T cells in severe patients, since a significantly greater number of CTLs responses are HLA-B restricted (65).

Thus, the results of our study strongly suggest a putative role of HLA-C genetic variation in the development of a specific immune response to COVID-19. The exact role of HLA-C*04:01 to the COVID-19 severity merits further study. However, screening for HLA-C genotype in patients from the populations, in which HLA-C*04:01 is a risk allele for the severe COVID-19, in addition to demographic variables, may support strategic clinical management of the disease.

## Data Availability Statement

The data presented in the study are deposited in the Figshare repository, with the following link https://figshare.com/projects/HLA_sequencing_data/130310.

## Ethics Statement

The studies involving human participants were reviewed and approved by Ethics Committee of Yerevan State Medical University after Mkhitar Heratsi (Meeting of 04.06.2020, Protocol N 7-3/20). Written informed consent to participate in this study was provided by the participants’ legal guardian/next of kin.

## Author Contributions

FJ initiated the study. VM designed and developed the project. AHo, KM, and LY performed the data analyses and interpretation of the results. KM provided data analysis methodology and the results conceptualization. ZM and RA performed ABMDR database data analyses. AHy, SA, MN, and AS collected the data. AHo and KM performed statistics, designed the figures and wrote the manuscript. All authors contributed to the final version of the manuscript.

## Funding

The research was supported in part by funds from Dr. George and Carolann Najarian, Armenian Medical Society of California. AnH and LY were supported by the 21AG-1F025 Grant from State Committee Science of the Ministry of Education and Science of the Republic of Armenia. AnH, LY, and KRM acknowledge the grant E17 provided by the State Committee Science of the Ministry of Education and Science of the Republic of Armenia.

## Conflict of Interest

RA and ZM declare that they work at the ClinSoft Armenia, Yerevan, Armenia, and ClinStat Group, Lexington, MA.

The remaining authors declare that the research was conducted in the absence of any commercial or financial relationships that could be construed as a potential conflict of interest.

## Publisher’s Note

All claims expressed in this article are solely those of the authors and do not necessarily represent those of their affiliated organizations, or those of the publisher, the editors and the reviewers. Any product that may be evaluated in this article, or claim that may be made by its manufacturer, is not guaranteed or endorsed by the publisher.
