# Supplementary figures and images for: HLA-C*04:01 Affects HLA Class I Heterozygosity and Predicted Affinity to SARS-CoV-2 Peptides, and in Combination With Age and Sex of Armenian Patients Contributes to COVID-19 Severity
Source: Front Immunol. 2022 Feb 3;13:769900. doi: 10.3389/fimmu.2022.769900 (PMC8850920; doi:10.3389/fimmu.2022.769900)

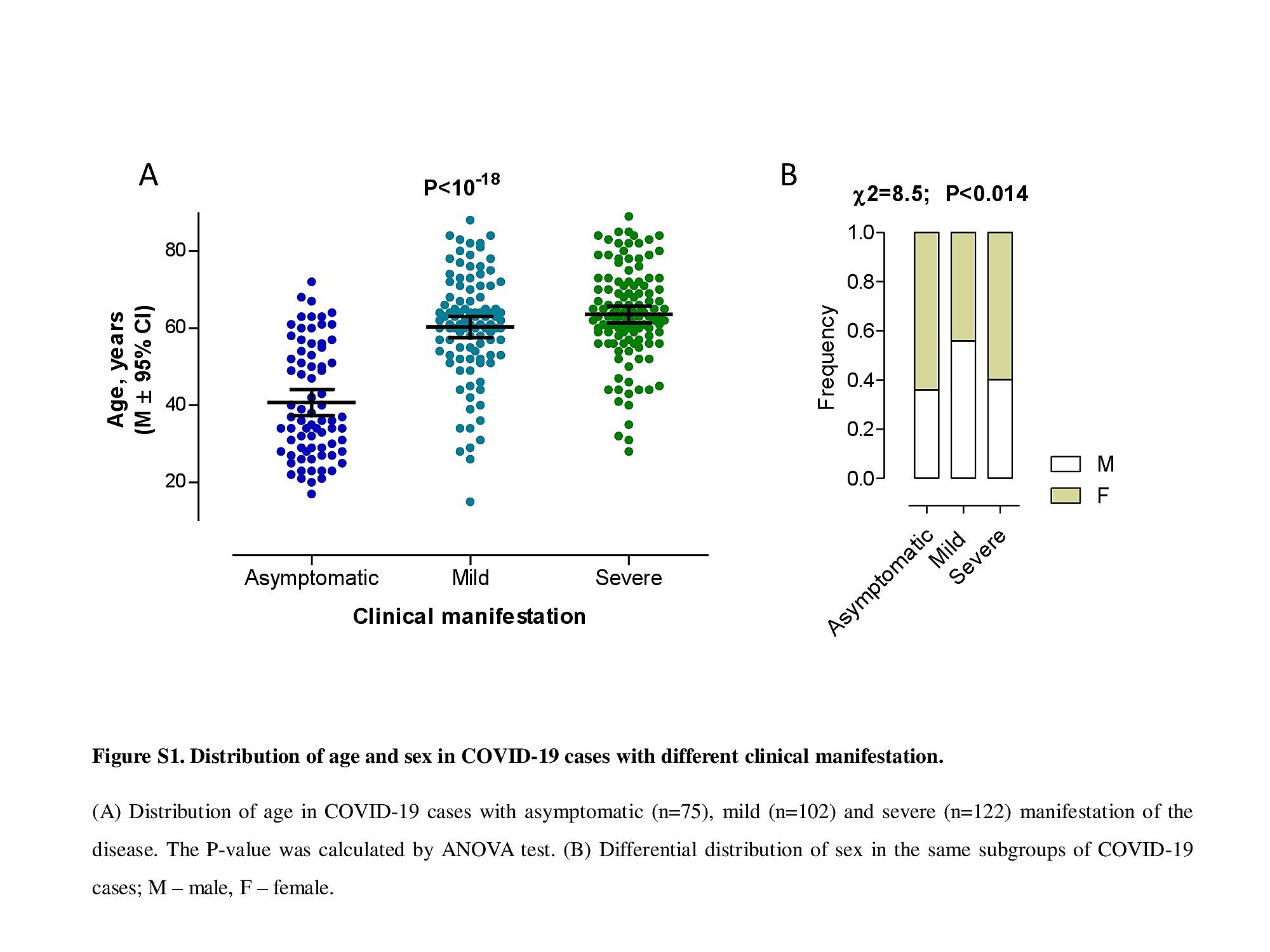

Supplement: Supplementary file 1 [file Image_1.jpeg]

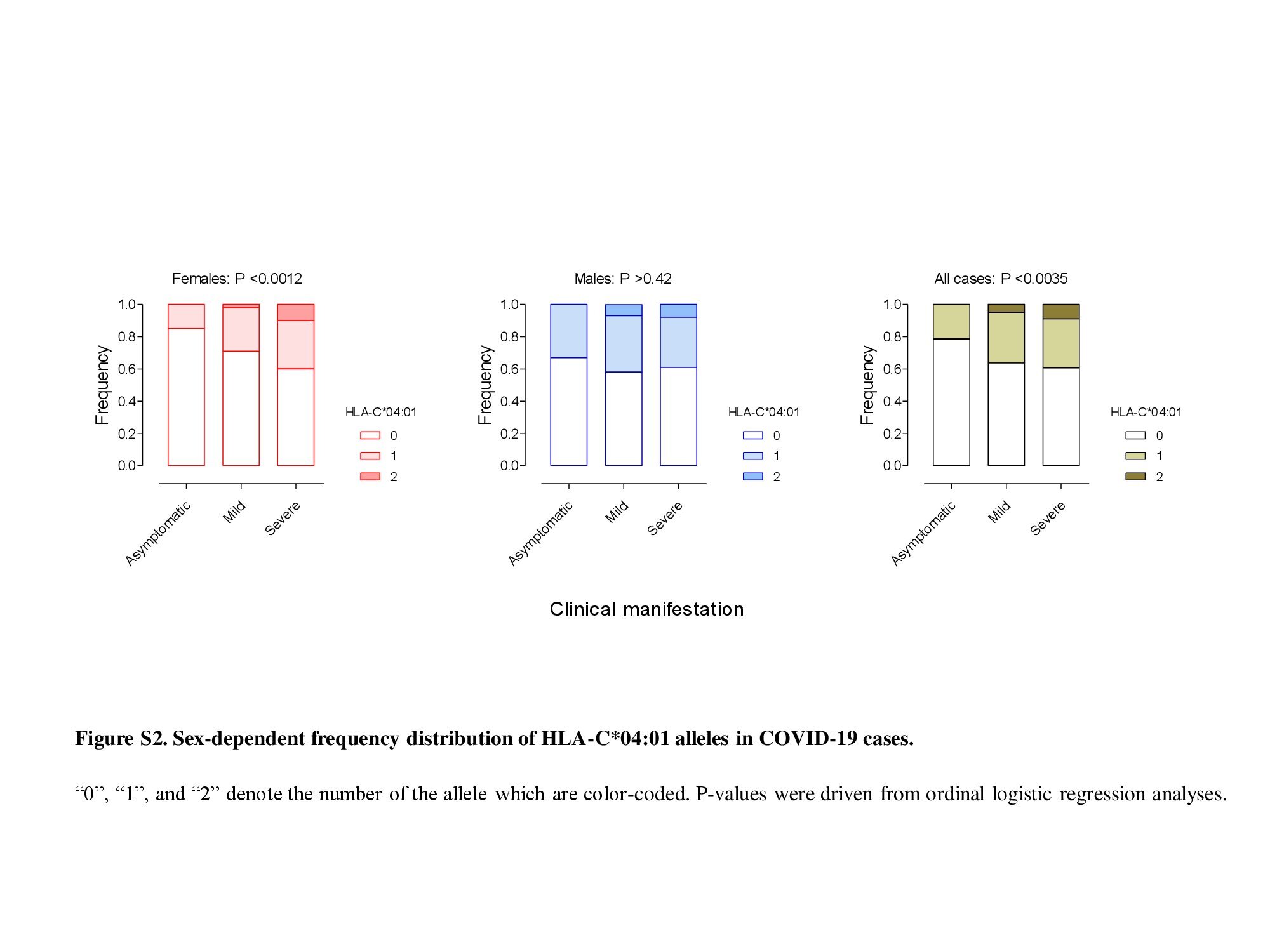

Supplement: Supplementary file 2 [file Image_2.jpg]

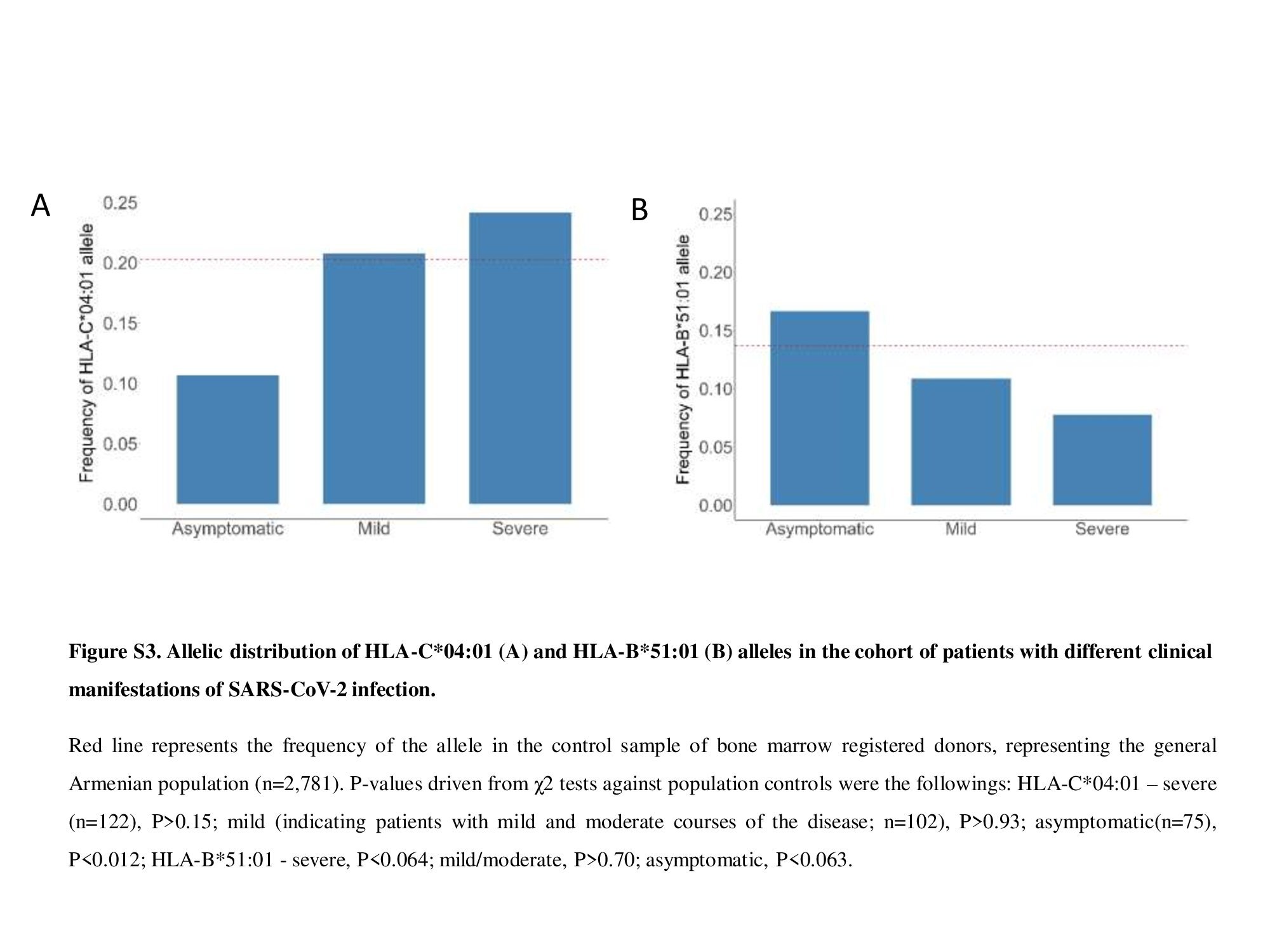

Supplement: Supplementary file 3 [file Image_3.jpg]

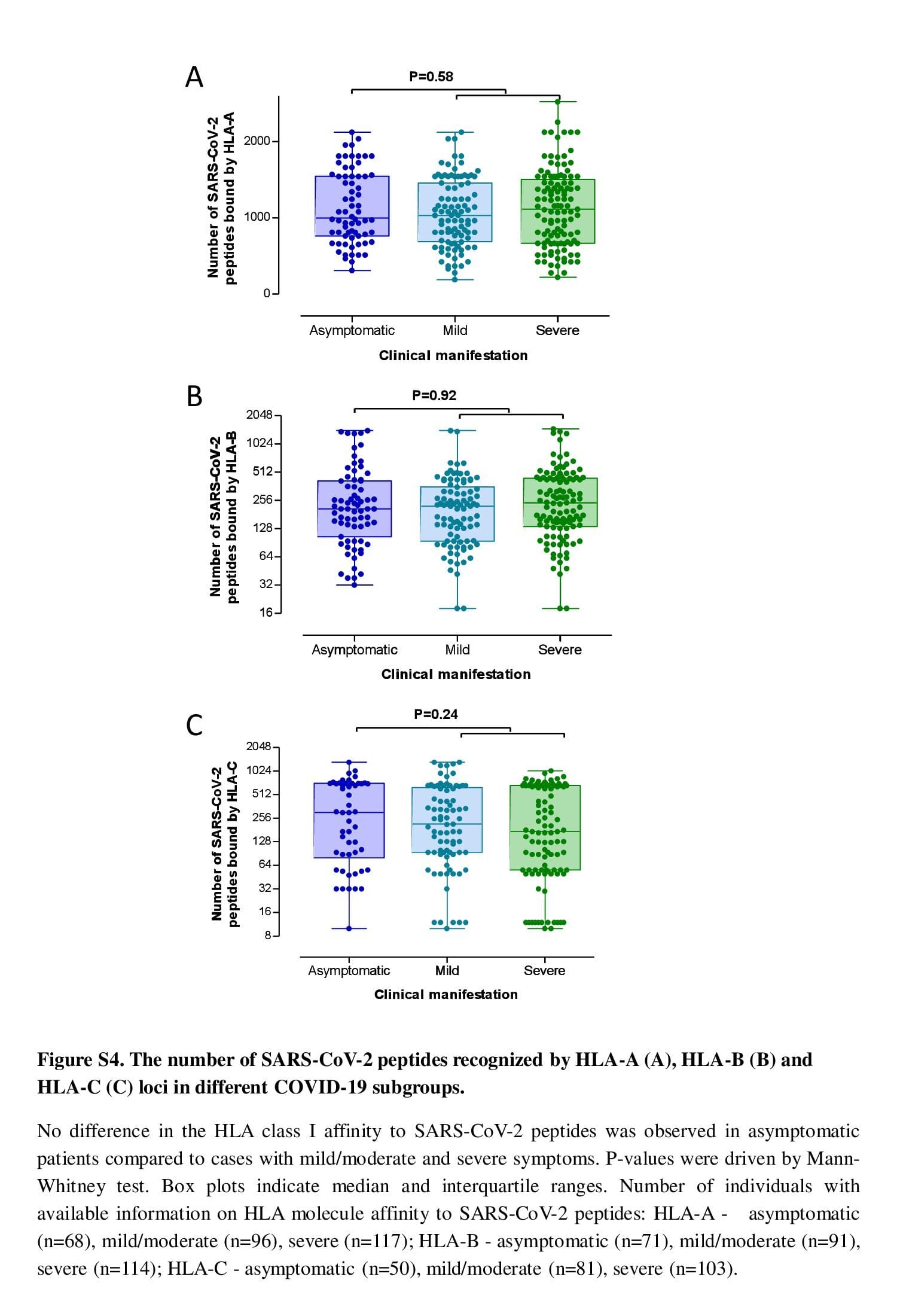

Supplement: Supplementary file 4 [file Image_4.jpg]

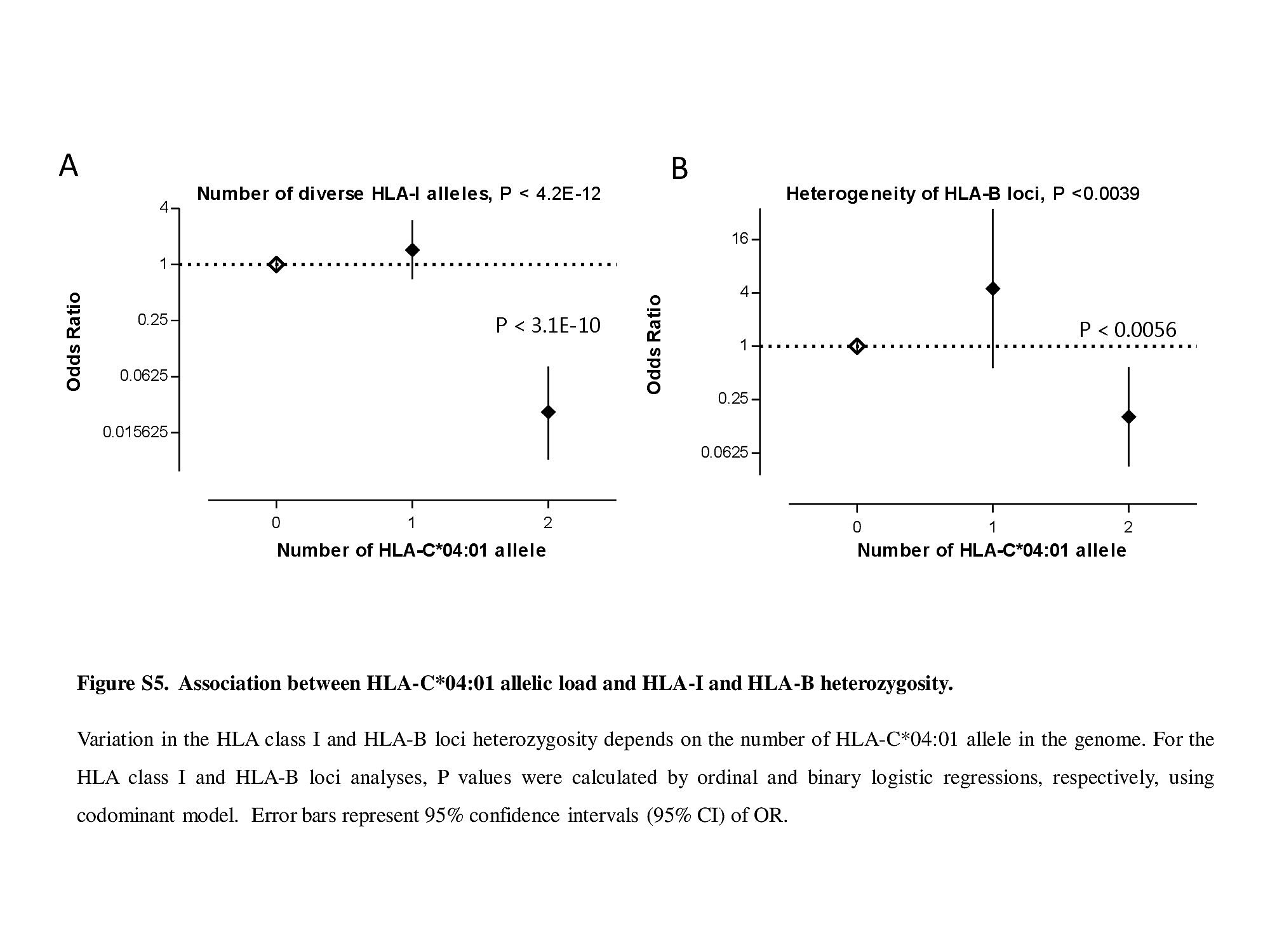

Supplement: Supplementary file 5 [file Image_5.jpeg]

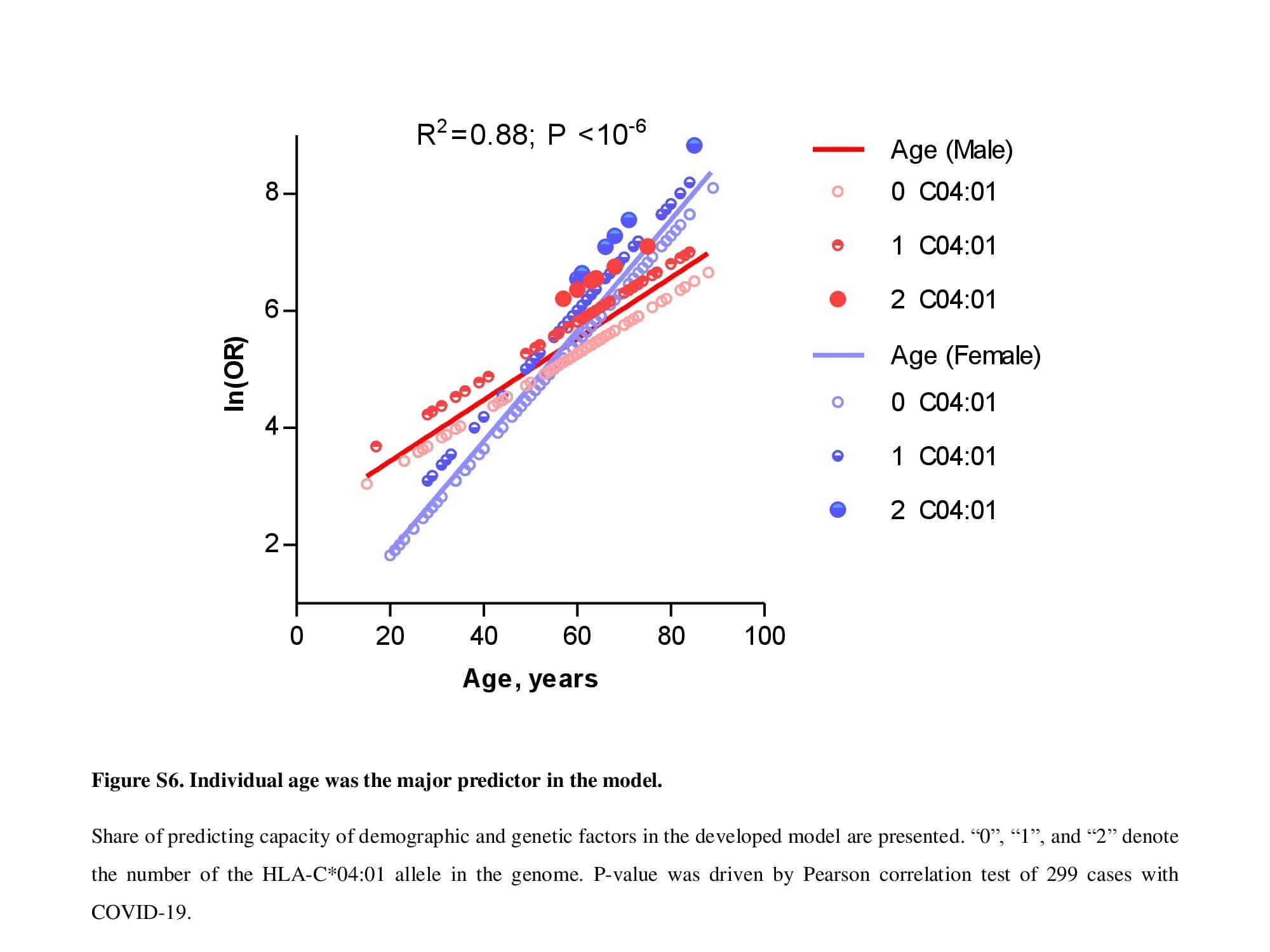

Supplement: Supplementary file 6 [file Image_6.jpg]

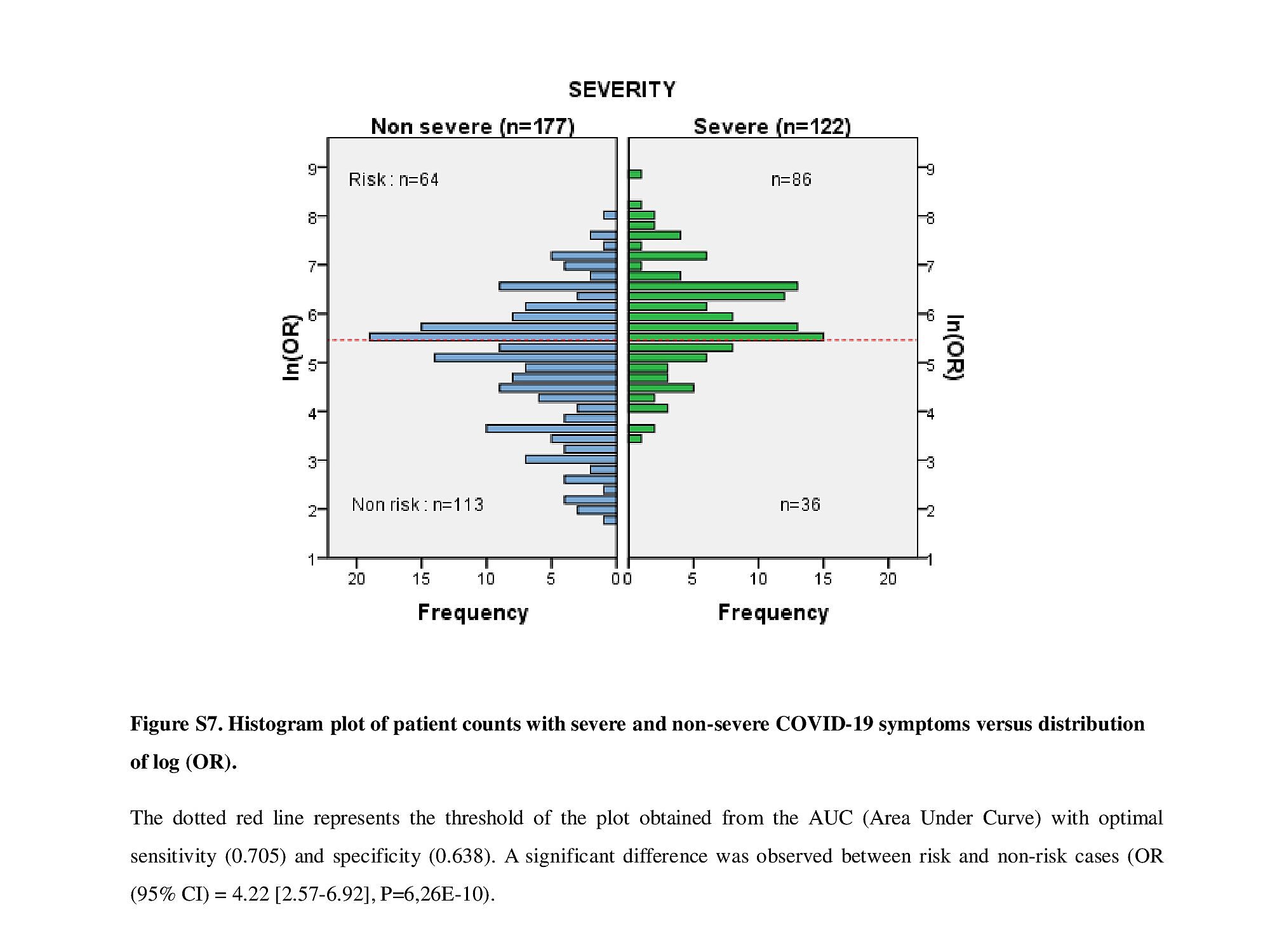

Supplement: Supplementary file 7 [file Image_7.jpeg]
